# The effect of touch simulation in virtual reality shopping

**DOI:** 10.1186/s40691-022-00312-w

**Published:** 2022-10-15

**Authors:** Ha Kyung Lee, Namhee Yoon, Dooyoung Choi

**Affiliations:** 1 Department of Clothing and Textiles, Chungnam National University, 99 Daehak-ro, Yuseong-gu, Daejeon, 34134 Republic of Korea; 2 Human Ecology Research Center, Korea University, 145 Anam-ro, Seongbuk-gu, Seoul, 02841 Republic of Korea; 3 Department of STEM Education and Professional Studies, Old Dominion University, Norfolk, VA 23529 USA

**Keywords:** Virtual reality (VR), VR store, Touch simulation, Self-efficacy, Pleasure, VR store satisfaction, Need for touch (NFT), Autotelic need for touch, Instrumental need for touch

## Abstract

This study aims to explore the effect of touch simulation on virtual reality (VR) store satisfaction mediated by VR shopping self-efficacy and VR shopping pleasure. The moderation effects of the autotelic and instrumental need for touch between touch simulation and VR store satisfaction are also explored. Participants wear a head-mounted display VR device (Oculus Go) in a controlled laboratory environment, and their VR store experience is recorded as data. All participants’ responses (n = 58) are analyzed using SPSS 20.0 for descriptive statistics, reliability analysis, exploratory factor analysis, and the Process macro model analysis. The results show that touch simulation positively influences VR store satisfaction, which is mediated by the self-efficacy and by the dual path of the self-efficacy and the pleasure. Furthermore, the relation between touch simulation and pleasure is moderated by need for touch. For individuals with a high level of autotelic need for touch, the effect of touch simulation on the pleasure is heightened. However, instrumental need for touch does not moderate the path of touch simulation on the self-efficacy.

## Introduction

The COVID-19 pandemic outbreak has changed the way the world socializes. Social activities have shifted from offline face-to-face interactions to online and virtual modes, and lockdowns, owing to the pandemic, forced people to turn to technology and amplified the merits of virtual environments. Virtual reality (VR) technology, a computer-generated interface simulating a realistic environment (Zheng et al., 1998), has become less distant and abstruse in people’s daily lives; it has marked its presence among the general public. With the aid of VR technology, people can travel to national parks, go underwater, engage in cycling at international scenic routes, and take a boxing class, all the while staying at home. VR technology also allows consumers to enjoy virtual shopping experiences without going to brick-and-mortar stores and provides solutions for the retail industry to maximize the marketing potential by shifting offline shopping to virtual online shopping because consumers cannot shop offline due to isolation and quarantine orders during the COVID-19 pandemic.

The pandemic has certainly emphasized the benefits of VR, although several pioneering retailers had already begun using various versions of the VR experience. Topshop offered a live virtual fashion show experience during the London Fashion Week in 2014. For the launch of its James Harden’s signature basketball sneakers, Adidas installed an in-store VR experience that allowed customers to hear and feel the floor rumbling and the wind blowing as Harden dribbled down the court (Burns, 2016). Lancôme, the luxury cosmetics brand of L’Oréal, opened its full-scale VR simulation of a physical store wherein shoppers can interact with the merchandise and sales representatives in the VR store (ByondXR, 2021). With the use of VR technology, retailers can not only offer novel digital experiences to consumers but also gain practical advantages, such as unlimited opening hours similar to online stores, while providing a sense of *being in a store* similar to shopping in a physical store. As seen in these examples, VR technology can be used in physical and online retail settings. We focus on VR stores that are virtual spaces or 3D digital environments where a physical store space is mimicked through VR technology (Pizzi et al., 2019), allowing people to be immersed in a fully digitalized environment by wearing a headset or a head-mounted device.

Given the emergence and importance of VR as a promising technology in the shopping and retail context (Cowan & Ketron, 2019; Moes & van Vliet, 2017), research on the use of VR technology in shopping is rapidly increasing (Xi & Hamari, 2021). After reviewing 72 studies that examined VR application to the shopping context, Xi and Hamari (2021) identified two broad categories that have been explored in relation to consumers’ behavioral outcomes: product-related and system-related factors. Product-related factors include various product presentations, such as 360° and three-dimensional images (Martínez-Navarro et al., 2019), product types, and product information (Ketelaar et al., 2018; Peukert et al., 2019; Zhao et al., 2017). System-related factors include the characteristics of VR environments identified by comparing the differences between VR and physical (Lombart et al., 2020; van Herpen et al., 2016; Waterlander et al., 2015) or online environments (Hsu et al., 2020) and examining the degrees of immersion driven by various types of hardware, such as head-mounted displays, body-tracking sensors, and motion-tracked controllers that enable walk-around navigation (Alshaer et al., 2017; Gorini et al., 2011; Lee & Chung, 2008; Shin & Shin, 2011). Similar to immersions, researchers have also explored other determinants that induce virtual experience for consumers, such as vividness and interactivity (Hur et al., 2019; Kang et al., 2020; Kim et al., 2021; Park et al., 2018; Violante et al., 2019), impressiveness (Peukert et al., 2019; Violante et al., 2019), and customization/personalization (Elboudali et al., 2020; Lau & Ki, 2021; Shuai et al., 2018).

In addition to the product- and system-related factors that influence consumers, consumers’ psychological aspects related to VR shopping have also been explored (Xi & Hamari, 2021). Some studies have investigated the mediating role of cognitive aspects, including perceived value/benefits (Altarteer & Charissis, 2019; Farah et al., 2019; Huang et al., 2019), memory recall (Bramley et al., 2018; Liang et al., 2019; Martínez-Navarro et al., 2019), and information process/attention (Kang et al., 2020; Siegrist et al., 2019; Violante et al., 2019). Other researchers have studied consumers’ perceived positive emotions (Israel et al., 2019; Jin et al., 2021; Kang et al., 2020; Peukert et al., 2019; Violante et al., 2019) and perceived realism (Carlson et al., 2011; Meißner et al., 2019; Schnack et al., 2019).

Although an increasing number of research studies on VR shopping have been conducted, the focus has been relatively limited to exploring the performance of VR shopping driven by its technological characteristics (e.g., vividness, interactivity, customization, and personalization). Accordingly, further discussions on VR shopping driven by consumer characteristics are necessary. To help close the gap in the literature, the current study examined consumer characteristics and consumers’ psychological responses enabled by VR technology as follows. First, this study adopts the concept of touch simulation to explore the psychological mechanism that underlies individuals’ attraction to VR stores. Touch simulation refers to the mental imagery of touching a product or imaging haptic attributes (Lee & Choi, 2021). If consumers’ touch simulation can be activated in the VR shopping environment, then consumers are likely to experience a feeling of touch for the merchandise in their mind (e.g., texture and hardness). Because a proximate feeling of texture and sensory experience through mental simulation can influence consumer decisions (Elder & Krishna, 2012; Lee & Choi, 2021; Liu et al., 2019; Shen et al., 2016), the exploration of touch simulation in VR stores can make a meaningful contribution to the literature considering that research on the effect of sensory experience on consumer responses in a VR environment remains limited, particularly in the context of the vicarious tactile experience.

Second, this study explores the mediating effect that may influence the effect of touch simulation on consumers: individuals’ VR shopping self-efficacy as a utilitarian path and VR shopping pleasure as a hedonic path. The application of utilitarian and hedonic factors has been widely adopted in the understanding of consumer behaviors which include online behaviors (e.g., To et al., 2007). Specifically, Abdullah and Ward (2016) conducted a meta-analysis where a utilitarian path through self-efficacy and an experiential path through enjoyment and experience to e-learning adaptation (i.e., learning through computer network technology, such as the Internet) were highlighted. Considering that e-learning and VR shopping can be viewed in the same perspective that users should accept new technologies to perform their own tasks, this study proposes self-efficacy and pleasure as the dual paths that consumers take for VR shopping, representing utilitarian and hedonic factors. Furthermore, we confirm this dual mediating path (i.e., through self-efficacy and pleasure) by examining the moderating effects of the two types of *need for touch*; the instrumental need for touch as a utilitarian motive (i.e., consumers’ touch preference to gain information about products from a utilitarian perspective) and the autotelic need for touch as a hedonic motive (i.e., consumers’ touch preference to seek for a source of pleasure and sensory enjoyment). As two types of need for touch can explain how individuals use tactile information as a source of utilitarian and hedonic desires (Kergoat et al, 2012), instrumental and autotelic need for touch can strengthen the mediating roles of self-efficacy and pleasure as utilitarian and hedonic paths for VR shopping experience. With the findings from this study, we aim to bridge the academic gap in the literature on why consumers derive satisfaction from VR shopping by clarifying the roles of touch simulation and related mediating variables of VR shopping self-efficacy and pleasure.

## Literature review

### Touch simulation

The formation of mental imagery of hypothetical realities is called mental simulation (Markman et al., 2009). Mental simulation, the enactment of a perceptual experience (Barsalou et al., 2003), can occur unintentionally and be automatically triggered by exposure to representations of an object, such as an image of the object. The enacted experience is based on previous experiences. For example, when a person looks at a leather jacket, the brain extracts the diverse sensory cues associated with that leather jacket (e.g., how it appears visually, what its texture feels like when touched, or how it has a particular smell). Later, when the person is exposed to a picture of a leather jacket, the earlier perceptions relating to the leather jacket are simulated, resulting in the activation of the same regions in the brain that were activated during the actual experience. Because of the activation in the brain, people can have perceptual experiences from a picture without having the actual object in hand. Like this, several studies have reported the effects of product visuals on the facilitation of consumer’s mental simulation, which in turn, influences purchase intention. Specifically, Elder and Krishna (2012) found that due to the ease of mental simulation from previous experiences, seeing a product picture that is oriented toward one’s dominant hand (e.g., a hamburger grabbed with the right hand) enhanced the mental imagery of reaching for the product and resulted in increased intention to purchase the product.

Similar to the proper orientation of a product that facilitates viewers’ mental simulation, the quality and the size of product visuals also enhance one’s mental simulation for interacting with a product (MacInnis & Price, 1987; Percy & Rossiter, 1983; Song & Kim, 2012). The clear presentation of a product providing more information about the product can stimulate a rich sensory experience (Jiang & Benbasat, 2007; Mann et al., 2015). A graphical demonstration of a product in a virtual environment makes it easier for people to imagine the product more vividly. Even with a two-dimensional product image, MacInnis and Price (1987) found that image processing is facilitated and consumption images are more stimulated when products are presented in larger sizes rather than smaller. Therefore, it is important to make it easy for consumers to imagine the product in reality because it can alleviate the perception of risk when they are unable to see or touch the actual product (Laroche et al., 2005).

Besides the effect of images, individuals’ actions or movements can also activate mental simulation (Barsalou 2008; Liu et al. 2019; Shen et al., 2016). Although no consumers would touch a flat screen over a product image to feel the texture of a product, the hand motion that is required to navigate a touch device over the product image results in facilitating the consumer’s touch simulation. Several studies have demonstrated that hand motions of touching a product image facilitated the feeling or simulation of actual actions (i.e., touching the product in reality), which evoke consumers to imagine the haptic attributes of a product (e.g., texture, hardness, temperature) (Liu et al., 2019). When the effects of direct touch through touchscreen devices were compared with the effects of non-touch devices, such as a laptop using a mouse, a greater mental simulation for touch was reported when using a touchscreen device rather than a laptop (Lee & Choi, 2021; Shen et al., 2016). This was because touching the product image activated individuals’ prior perceptions relating to the product and led to an experience of seemingly touching the product. Such facilitated touch simulation led to positive consumer responses, such as a favorable attitude toward the product and a higher purchase intention.

### Touch simulation in VR environment

Touch simulation in VR environments can be explained by construal level theory (CLT, Trope & Liberman, 2003). According to CLT, information can be construed as either a high or low level on the basis of its degree of abstractness or concreteness. A high-level construal is an abstract mental representation, whereas a low-level construal is a relatively concrete mental representation and contains additional detailed information. The distinction among construal levels can be determined by psychological distance (Trope & Liberman, 2010). Psychologically distant (vs. proximal) subjects lead to a high (vs. low) construal level, and high-level construal can reciprocally induce a further psychological distance (Bar-Anan et al., 2006).

In a sensory-rich VR environment, consumers can experience a high degree of presence (being in there) through VR stimuli, such as the senses of sight, hearing, and even touch, depending on the technology (Cahalane et al., 2022). Sensory experiences in VR can be further enhanced through a high degree of interactivity by using a controller or a device to experience the interface (Shu et al., 2019). In accordance with CLT, these VR characteristics may reduce the psychological distance between users and virtual products. Thus, psychologically proximal products in VR shopping can induce concrete mental simulation, including touch simulation.

The concrete mental representation driven by a low-level construal can have significant consequences on consumers’ affect, cognition, evaluation, and decision-making process (Freitas et al., 2004; Labroo & Lee, 2006; Park & Morton, 2015; White et al., 2011). Furthermore, just like how touch simulation generates positive consumer responses (e.g., Elder & Krishna, 2012; Lee & Choi, 2021; Liu et al., 2019; Shen et al., 2016), we expect to see similar positive consumer responses through the facilitated mental simulation for touch in a VR store. Specifically, in this study, the effect of touch simulation in a virtual environment is measured by VR store satisfaction. VR store satisfaction has been explored in previous research, and the results have revealed that VR-based stores generate not only a similar level of store satisfaction as that of a physical store (Pizzi et al., 2019) but also a greater purchase intention when compared with two-dimensional video product presentations (Van Kerrebroeck et al., 2017). One reason for this could be attributed to VR technology which helped shoppers gain a memorable shopping experience, thus eliciting higher consumer satisfaction (Srinivasan & Srivastava, 2010). We propose that the touch simulation enabled by VR technology is another reason for the positive consumer satisfaction with a VR store. Considering previous studies on positive consumer responses resulting from touch simulation (e.g., Elder & Krishna, 2012; Lee & Choi, 2021; Liu et al., 2019; Shen et al., 2016), we expect to see touch simulation in a VR environment leading to VR store satisfaction, thereby proposing the first hypothesis as follows:

#### H1.

Touch simulation in VR stores positively influences VR store satisfaction.

### VR shopping self-efficacy

Like any other technology innovation adoption, the acceptance and use of VR technology vary among individuals. One factor that may influence the adoption of VR shopping technology is consumer self-efficacy in using the technology. Self-efficacy refers to personal judgments of one’s ability to arrange and execute actions in specific situations with novel or unpredictable features (Bandura, 1977). Self-efficacy has been applied across diverse research areas such as psychology (Ajzen, 2002; Bandura, 1977; Schunk, 1984), education (Schunk, 1984), consumer behavior (Ellen et al., 1991; Hill & Beatty, 2011; Meuter et al., 2005), advertising (Manyiwa & Brennan, 2012), and information technology, such as the use of computers (Compeau & Higgins, 1995). Because self-efficacy is based on individuals’ belief in themselves for performing an action (Kulviwat et al., 2014), the concept of self-efficacy can be applied to diverse actions in various contexts, such as using new technology for shopping.

Drawing from self-efficacy theory, researchers examined online-related self-efficacy, which can be applied to self-efficacy in the use of VR shopping technology. The three types of self-efficacy relevant to the use of VR shopping technology are online consumer self-efficacy (Moschis & Moore, 1978), internet self-efficacy (Wei & Zhang, 2008), and mobile shopping self-efficacy (Chiu et al., 2011; Lu & Su, 2009; Zhou et al., 2016). Online consumer self-efficacy refers to individuals’ perception of their capability to engage effectively as a shopper in the online marketplace (Moschis & Moore, 1978). Hill and Beatty (2011) addressed two dimensions of online consumer self-efficacy: (1) online shopping self-efficacy related to general online shopping knowledge, such as knowing how to search for product prices on the Internet, and (2) online technical self-efficacy relating to technical ability such as the ability to navigate online websites. The researchers demonstrated that online shopping involvement and online usage experience are the antecedents of online consumers’ self-efficacy.

Internet self-efficacy is a broader concept than online consumer self-efficacy, referring to the judgment/evaluation of one's capability to use the Internet in general (Wei & Zhang, 2008). It captures the idea of confidence in accomplishing tasks on the Internet. Similarly, mobile shopping self-efficacy refers to the perceived ability of individuals to use mobile devices in searching for useful information, placing orders, and handling unexpected problems with transactions (Zhou et al., 2016). Based on the concept of self-efficacy in the field of online and mobile shopping, we define self-efficacy in VR shopping, that is, VR shopping self-efficacy, as the perceived ability and confidence in shopping relevant behaviors in VR stores, such as browsing for products, obtaining relevant information, and choosing the appropriate product.

Studies of self-efficacy have some evidence that can explain the relationship between touch simulation and VR shopping self-efficacy. In the original proposal of the self-efficacy theory, Bandura (1977) identified four sources of high self-efficacy which include vicarious experiences, such as seeing others perform activities. Schunk (1984) concurred with Bandura and confirmed the sources of self-efficacy in understanding people’s achievement behaviors. Also, research has particularly noted that the imagery of success through vicarious experience has the most direct and instant effect on self-efficacy (Bandura, 2000; Harlow et al., 2006; Lane et al., 2004; Prieto & Meyers, 1999). The vicarious experiences, experiencing secondhandedly or in the imagination, correspond with the consumer’s experience of touch simulation because touch simulation is a type of vicarious experience through mental imagery of touch. Accordingly, the effects of vicarious experience on self-efficacy can be applied to touch simulation and VR shopping self-efficacy. Through vicarious touch experience in a VR store, consumers can gather information about a product and be motivated to perform their VR shopping successfully, and thus, consumers’ VR shopping efficacy can be positively influenced. The more the touch simulation consumers experience, the higher their VR shopping self-efficacy.

The link between touch simulation and self-efficacy can also be explained by Mehrabian and Russell’s stimulus–organism–response (SOR) model (1974), wherein a sense of touch (stimulus) elicits a psychological reaction (organism) leading to consequences such as product or store evaluation (response). In prior literature, the self-efficacy of individuals was treated as a psychological organism in the context of the SOR paradigm (Attiq et al., 2017). Thus, we hypothesize the following:

#### H2.

Touch simulation in VR stores positively influences VR shopping self-efficacy.

Self-efficacy influences not only individuals’ self-motivation in stressful situations but also their evaluative judgments or attitudes toward a situation or an action (Bandura, 1977; Compeau & Higgins, 1995), which includes new technology adaption (Ellen et al., 1991; Sinkovics et al., 2002). Considering the relatively unfamiliar and technically challenging terrain of VR technology, VR shopping self-efficacy is expected to play a significant role in the VR shopping experience and VR store satisfaction. Previous research also demonstrated that one’s attitudes or behavioral intentions were predicted by self-efficacy (Ryan & Deci, 2000; Wang, 2015). Individuals with high (vs. low) self-efficacy likely believe that the consequence of a behavior is good; thus, they can show a high level of positive attitude (Wang, 2015).

According to self-determination theory (Ryan & Deci, 2000), if individuals think that they have the ability or confidence to perform a task (e.g., self-efficacy for shopping in a VR store), then this confidence enhances their intrinsic motivation, leading to the belief that the outcomes of the task are good and satisfactory (Bandura, 2000), thereby increasing favorable attitudes. That is, consumers with high self-efficacy in VR shopping will be likely to use the VR shopping technology with more willingness and make more efforts in using the technology; they will also be less likely to feel anxious about VR shopping and more likely to perceive the VR shopping experience with ease. Accordingly, a high VR shopping self-efficacy could have a positive impact on consumer satisfaction in a VR store. On the contrary, consumers with a low level of self-efficacy in VR shopping would be less motivated to use the VR technology, less likely to make efforts to use the technology, and face a higher degree of anxiety and difficulties in navigating the VR store. Such lack of motivation and effort, combined with a high degree of anxiety and perceived difficulties, could negatively influence the consumer VR store experience, which in turn reduce the level of satisfaction obtained by the consumer in a VR store. Based on this, we propose hypothesis 3 as below:

#### H3.

VR shopping self-efficacy positively influences VR store satisfaction.

### VR shopping pleasure

Consumers enjoy using VR technology and VR environments not only for a new experience (Lee et al., 2019) but also for experiencing various emotional arousals through a high level of telepresence, which indicates users’ immersive experience of a computer-generated reality (Steuer, 1992; Weibel et al., 2008). Telepresence created by the VR environment enables consumers to experience the environment with their senses, and their engagement with the senses generates emotional responses (Lehtonen et al., 2005). The close relationship between emotions and senses experienced in a VR world is evident from our brain as the part that manages the experience in a virtual environment also deals with the processing of individual senses and emotions (Baumgartner et al., 2008). Therefore, the experience of touch, one of the five senses, through VR technology can generate emotional responses in a VR environment, and the touch experience can equally apply to vicarious touch experience through telepresence experience.

Although it was not specific to VR environments, empirical evidence of the relationship between touch simulation and emotions has also been reported. For example, Peck and Childers (2003) found that touch, and even anticipated touch, can increase positive emotional responses such as pleasure and enjoyment. Grohmann et al. (2007) also verified that the effect of touch on product evaluation is mediated by emotional responses (e.g., pleasure, arousal). Prior literature also indicated that sensory pleasure is derived from the physical sensation of holding, feeling, or touching a product (Cho et al., 2019) on the basis of the relationship between the stimulus and organism in the SOR model. In this regard, we hypothesize that vicarious touch through touch simulation influences individuals’ pleasure in a VR shopping environment. Pleasure enhanced by VR environments in turn increases satisfaction with a VR store according to the SOR paradigm (Grohmann et al., 2007; Jin et al., 2021; Mehrabian & Russell, 1974;). The following hypotheses are proposed based on the above:

#### H4.

Touch simulation in VR stores positively influences VR shopping pleasure.

#### H5.

VR shopping pleasure positively influences VR store satisfaction.

Self-efficacy has been considered an essential antecedent of positive emotions such as playfulness, fun, and pleasure (Csikszentmihalyi, 1975; Kulviwat et al., 2014; Webster & Martocchio, 1992). Particularly, Kulviwat et al. (2014) found that self-efficacy with regard to a specific technological innovation increased pleasure, arousal, and dominance in adopting a high-tech product; consumers tend to enjoy using high-tech products with greater confidence and comfort. Compeau and Higgins (1995) also revealed the relationship between individuals’ self-efficacy and enjoyment in the adoption of new technology. These results can be explained by the theory of optimal flow (Kulviwat et al., 2014), whereby the necessary skills for achieving a difficult and demanding task enhance one’s positive emotions about the task (Csikszentmihalyi, 1990; Hoffman & Novak, 1996). When people believe that they can perform specific tasks, they tend to enjoy such behaviors. Thus, we postulate that self-efficacy in VR shopping enhances individuals’ pleasure during VR shopping and propose the following:

#### H6.

VR shopping self-efficacy positively influences VR shopping pleasure.

### The moderating effect of the need for touch between touch simulation and consumer responses

The effect of touch simulation can vary according to users’ urge to touch. Individual differences in desiring to touch is termed the *need for touch (NFT)*, and it is defined as “a preference for the extraction and utilization of information obtained through the haptic system” (Peck & Childers, 2003, p. 431). Peck and Childers (2003) conceptually categorize NFT into two types: autotelic and instrumental. The autotelic NFT refers to the hedonic desire for touch. People with high autotelic NFT seek sensory stimulation from touching. They touch for fun and enjoyment, and their touch is compulsive (Peck & Childers, 2003). On the contrary, the instrumental NFT refers to the utilitarian desire for touch. For them, touch is purposeful because people with high instrumental NFT use touch to evaluate product properties and judge the product (Peck & Childers, 2003).

Researchers have reported different consumer responses based on the NFT type. People with high autotelic NFT reported higher emotional responses from touch (Peck & Wiggins, 2006), showed stronger attachment to the product, and perceived the brand of the product as exciting when presented with incongruent haptic cues (i.e., heavyweight with smooth texture) rather than with congruent haptic cues (i.e., lightweight with smooth texture) (Atakan, 2014; Ranaweera et al., 2021). Further, compared to those with low instrumental NFT, people with high instrumental NFT reported a greater increase in their perception of the ease of using a product after touching it, which led to a higher purchase intention.

In both types of NFT, consumers with high NFT would place higher importance on the touch simulation, which enhanced their overall experience of the virtual environment. However, the influential role of touch simulation could vary by the type of NFT because the instrumental function of touch acts as a means of getting information on the products while the autotelic function of touch serves as a source of pleasure and sensory enjoyment (Kergoat et al., 2012). The relationship between touch simulation and VR shopping self-efficacy can be considered a utilitarian path because a vicarious touch experience can provide task-relevant information, which can empower one’s ability to use the product. On the contrary, the relationship between touch simulation and pleasure takes the hedonic path. Therefore, we postulate the two paths; individuals with high instrumental NFT will strengthen the utilitarian path for the effect of touch simulation on self-efficacy in a VR environment. Individuals with high autotelic NFC, who use tactile information as a source of pleasure, influence the relationship between touch simulation and VR shopping pleasure. Thus, we hypothesize as follows:

#### H7a.

The effect of touch simulation on VR shopping self-efficacy is stronger when individuals’ instrumental NFT is high (vs. low).

#### H7b.

The effect of touch simulation on VR shopping pleasure is stronger when individuals’ autotelic NFT is high (vs. low).

### The conceptual model

**Fig. 1:**
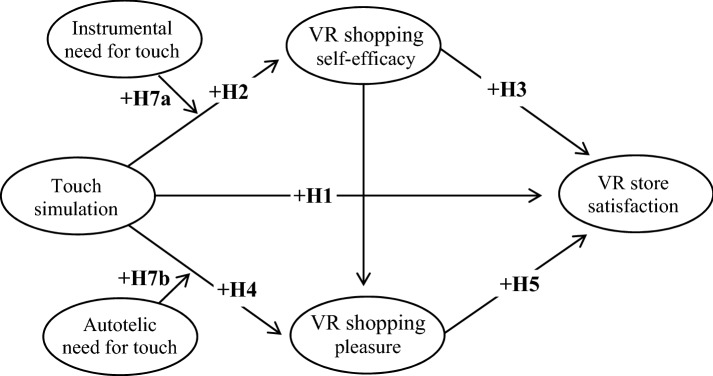
The conceptual model

On the basis of the proposed hypotheses, we form a conceptual model showing the effects of touch simulation in a VR store on VR shopping self-efficacy and VR shopping pleasure, which positively influence consumers’ VR store satisfaction (Fig. 1). The research model also includes the moderation effects of instrumental and autotelic NFT among consumer responses in a VR store (i.e., the mediating path of VR shopping self-efficacy and VR shopping pleasure on VR store satisfaction).

## Methods

### Procedure and stimulus

To test the hypotheses, a commercial VR store (m.lotteimall.com) was chosen as the stimulus. Among the existing VR stores of South Korea’s biggest retailers, we chose a VR store belonging to a fashion brand called *Saint James*. This VR store enabled users to explore the store in three-dimension with a 360-degree view through a mobile app or a VR device. The VR store presented VR products in clear images close to the size of those in the actual store and offered product information when consumers tapped on a tagged product.

Participants in this experiential laboratory study wore an Oculus Go, a head-mounted device VR device, in a controlled laboratory environment to create an immersive VR shopping experience. Only devices for the immersive VR experience were placed in the laboratory, and the laboratory condition was kept consistent across participants. To ensure a sufficient immersive VR experience, participants were individually invited to the laboratory. Oculus Go was also connected to a one-handed grip controller. Users could move the control stick by hand and feel the sensation of touching a product by pressing on the controller.

Participants were recruited through an announcement on the online bulletin board of a national university in South Korea. The data collection was conducted individually in the following manner. Participants were instructed to follow the same procedures and trained to use VR devices smoothly. First, participants were asked to respond to a questionnaire regarding their prior VR store experiences. All participants had no shopping experience at VR stores. Thus, samples were unfiltered for collecting data, and the procedure was consistently performed for users. Second, participants wore the headset and joysticks, and a researcher explained how to use the head-mounted device and search for products in VR environments. By following directions, participants wearing Oculus Go stood up, moved their heads and bodies, and walked around along the points in the VR store. They spent some time getting used to operating the device. Last, participants visited the stimulus VR store and explored it for as long as they wanted. They stayed for 2 to 8 min. After browsing the VR store, they were asked to respond to a questionnaire about touch simulation in the VR store, VR shopping self-efficacy, VR shopping pleasure, VR store satisfaction, and instrumental and autotelic NFTs.

### Measurement and participants

Touch simulation was measured by three items adapted from Shen et al. (2016). The items were: *I could imagine touching the product, I felt like touching the product,* and *I could imagine the texture of the product*. VR shopping self-efficacy was measured by five items adapted from the scale of online shopping self-efficacy by Hill and Beatty (2011). The items are: *I am very good at shopping for products at this VR store, I can easily shop at this VR store, I can shop in the environment I want at this VR store, I am sure I can shop for what I want at this VR store,* and *I think my shopping results at this VR store are very positive*. VR shopping pleasure was measured by three items adapted from Kulviwat et al. (2014): *Browsing at the VR store is interesting, VR shopping is a pleasant process,* and *It gives me pleasure to shop at the VR store*. VR store satisfaction was measured by three items (Poushneh & Vasquez-Parraga, 2017; Sands et al., 2015) which were *I like this VR store, I am satisfied with the VR store,* and *I find this VR store favorable*. The measures for instrumental and autotelic NFT were derived from Peck and Childers (2003) comprising 6 items; however, 2 items on autotelic NFT and 3 items on instrumental NFT were removed in this study based on factor loadings by exploratory factor analysis, which were lower than the criteria (> 0.60 threshold; Bagozzi & Yi, 1988). Instrumental NFT items were: *I place more trust in products that can be touched before purchase, I feel more comfortable purchasing a product after physically examining it,* and *I feel more confident making a purchase after touching a product* (The deleted three items were: *If I cannot touch a product in the store, then I am reluctant to purchase it. The only way to make sure a product is worth buying is to actually touch it,* and *There are many products that I will only buy if I can touch/hold them before purchase*)*.* The measurement items of autotelic NFT included were as follows: *When walking through the store, I can’t help touch all kinds of products, Touching products can be fun, I like to touch products even if I have no intention of buying them,* and *When browsing in stores, I like to touch lots of products* (excluding two items, namely, *When browsing in stores, holding all kinds of products is important for me,* and *I find myself touching all kinds of products in stores*). All measurement items were rated on a 5-point Likert scale ranging from 1 (strongly disagree) to 5 (strongly agree). Demographic questions were asked at the end of the questionnaire.

As the stimulus is a VR store for unisex casual clothing, subjects included female and male young adults in their 20 s and 30 s. Voluntary participants were recruited by convenience sampling, and a total of 58 participants completed the study. Participants consisted of 29 female (50%) and 29 male (50%) individuals, and their ages ranged from 20 to 37 years (Mean = 25.4 years). All participant responses were analyzed using SPSS 20.0 for descriptive statistics, frequency analysis, reliability analysis, and the PROCESS procedure.

Using a bootstrapping approach, the researcher can evaluate models varying from small to moderate sample sizes (i.e., 50 ~ 200) and moderate to large factor loadings (i.e., 0.60 ~ 0.90) on the basis of the idea that small samples with simple structures can compensate with strong factor loadings (Guadagnoli & Velicer, 1988; Nevitt & Handcock, 2001). Due to the nature of the experiential laboratory study, the sample size of the current study was quite small; thus, the PROCESS procedure for bootstrapping analysis was conducted.

## Results

### Measurement of reliability and validity

The reliability of the measurements was tested using Cronbach’s alpha. The measurement reliability results of touch simulation (α = 0.812), VR shopping self-efficacy (α = 0.920), VR shopping pleasure (α = 0.802), VR store satisfaction (α = 0.907), instrumental NFT (α = 0.793), and autotelic NFT (α = 0.852) were satisfactory; Cronbach’s alpha values for the composite reliability of the items ranged from 0.793 to 0.920 (> 0.70 threshold; Nunnally & Bernstein, 1994), thereby indicating that the internal consistency of all scales was statistically acceptable.

The values of the average variance extracted (AVE) of six constructs were over the threshold of 0.50 (Bagozzi & Yi, 1988), and it presented the satisfactory level of convergent validity of constructs. To determine the discriminant validity of the constructs, the AVE was compared with the squared correlation of other constructs (Table 1). All AVEs were greater than all the squared correlations (Fornell & Larcker, 1981), thus the level of discriminant validity was acceptable. Furthermore, the correlation coefficients of the main construct variables were significant in the range 0.361 to 0.685. Specifically, the correlation between touch simulation and pleasure was not significant (*r* = 0.126, *p* = 0.344). Each correlation between touch simulation and instrumental NFT and between autotelic NFT and pleasure was significantly negative. As moderators, instrumental NFT and autotelic NFT showed significant correlation (*r* = 0.332, *p* < 0.5).

**Table 1:** The results of the discriminant validity of the constructs

Variable	Mean (*SD*)	Cronbach’s α	1	2	3	4	5	6
1. Touch simulation	2.603 (0.755)	0.812	^1)^***0.836***	^3)^0.277	0.016	0.130	0.055	0.002
2. VR shopping self-efficacy	2.806 (0.819)	0.920	^2)^0.526^**^	***0.639***	0.146	0.469	0.006	0.006
3. VR shopping pleasure	3.402 (0.719)	0.802	0.126	0.382^**^	***0.650***	0.269	0.011	0.000
4. VR store satisfaction	3.425 (0.708)	0.907	0.361^**^	0.685^**^	0.519^**^	***0.582***	0.033	0.059
5. Instrumental NFT	4.327 (0.527)	0.793	− 0.235^**†**^	− 0.076	0.104	− 0.181	***0.678***	0.110
6. Autotelic NFT	3.745 (0.791)	0.852	0.044	− 0.077	0.003	− 0.244^**†**^	0.332^*^	***0.668***

^1)^Numerical value of diagonal: average variance extracted (AVE), ^2)^Numerical value of bottom of diagonal: correlation coefficient and ^3)^Numerical value of top of diagonal: squared correlation coefficient (*ϕ*^*2*^)

†*p* < 0.1, *p < 0.05, **p < 0.01

### Hypotheses test

To test the hypotheses, the mediating model of VR shopping self-efficacy and VR shopping pleasure between touch simulation and VR store satisfaction was analyzed by bias-corrected bootstrapping using Hayes (2018)’s PROCESS Macro Model 6 with 5000 samples. VR shopping time was controlled as a covariate. The confidence interval (CI) was set to 95%. The sequential mediation model was tested.

**Fig. 2:**
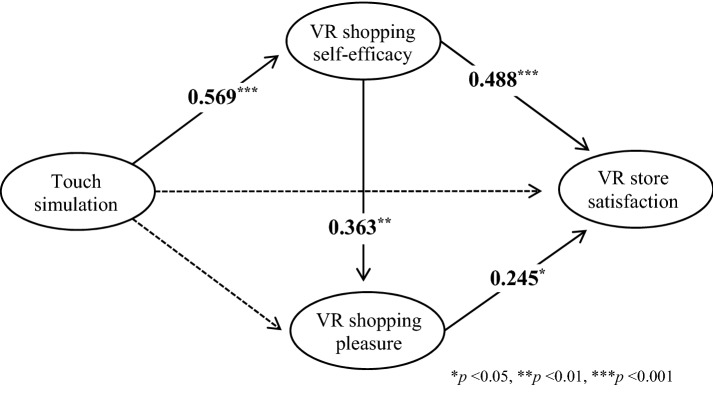
Mediation model for the effects of touch simulation on VR store satisfaction

First, as shown in Fig. 2, the direct effect of touch simulation as the independent variable on VR store satisfaction was not significant (Effect = 0.028, bootSE = 0.099, 95% CI [− 0.171, 0.228]), thereby rejecting H1. Second, the indirect effect of touch simulation on VR store satisfaction through VR shopping self-efficacy and VR shopping pleasure was significant (Effect = 0.278, bootSE = 0.078, 95% CI [0.131, 0.439]). Specifically, the effect of touch simulation on VR shopping self-efficacy was significant (*b* = 0.569, 95% CI [0.320, 0.818]). However, the effect of touch simulation on VR shopping pleasure was not significant (*b* = − 0.089, 95% CI [− 0.355, 0.176]). The direct paths of VR shopping self-efficacy (*b* = 0.488, 95% CI [0.289, 0.686]) and VR shopping pleasure (*b* = 0.245, 95% CI [0.041, 0.450]) on VR store satisfaction were statistically significant. The path of VR shopping self-efficacy on VR shopping pleasure was also significant (*b* = 0.363, 95% CI [0.117, 0.608]). Thus, H2, H3, H5, and H6 were supported, but H4 was rejected. The mediating path of VR shopping self-efficacy between touch simulation and VR store satisfaction was statistically significant (Effect = 0.278, bootSE = 0.078, 95% CI [0.131, 0.439]), and the indirect path through VR shopping pleasure was not significant (Effect = -0.021, bootSE = 0.041, 95% CI [− 0.125, 0.038]. The dual meditation path of VR shopping self-efficacy and VR shopping pleasure was also marginally significant (Effect = 0.050, bootSE = 0.036, 95% CI [0.003, 0.142]) (see Table 2). These results indicate that touch simulation affected VR store satisfaction, which was mediated by VR shopping self-efficacy and by the dual path of VR shopping self-efficacy and VR shopping pleasure.

**Table 2:** Indirect effects of touch simulation on VR store satisfaction

Path	Effect	BootSE	95% Confidence interval	
			LLCI^a^	ULCI^b^
Total	0.307	0.092	0.127	0.495
Indirect path 1	0.278	0.078	0.131	0.439
Indirect path 2	− 0.021	0.041	− 0.125	0.038
Indirect path 3	0.050	0.036	0.003	0.142

Indirect path 1: Touch simulation→VR shopping self-efficacy →VR store satisfaction

Indirect path 2: Touch simulation →VR shopping pleasure →VR store satisfaction

Indirect path 3: Touch simulation →VR shopping self-efficacy →Pleasure VR store satisfaction

^a^LLCI: lower limit confidence interval

^b^ULCI: upper limit confidence interval

**Fig. 3:**
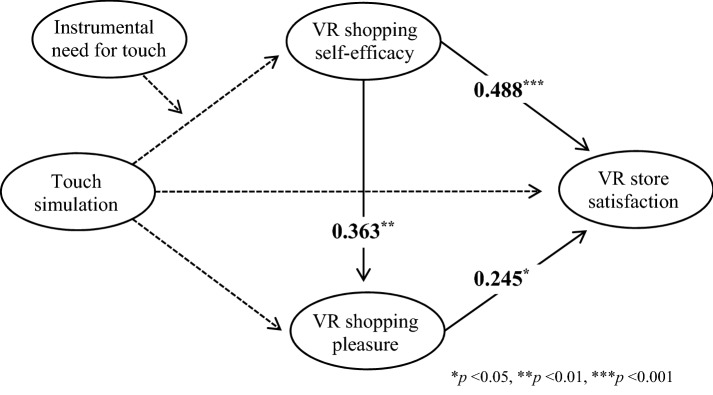
Moderating model of instrumental need for touch

To test H7a, the moderating role of instrumental NFT between touch simulation and VR shopping self-efficacy, the PROCESS Macro Model 83 was used with 5,000 bootstrap samples. VR shopping time was controlled as a covariate. As shown in Fig. 3, the interplay effect of touch simulation and instrumental NFT on VR shopping self-efficacy was statistically not significant (*b* = -0.138, 95% CI [-0.666, 0.389]). Although the indirect effect of touch simulation on VR store satisfaction through VR shopping self-efficacy and VR shopping pleasure was significant, as consistent with the previous result, the moderated mediation effect of instrumental NFT was not significant (index of moderated meditation = -0.067, 95% CI [− 0.383, 0.176]). Thus, H7a was rejected.

**Fig. 4:**
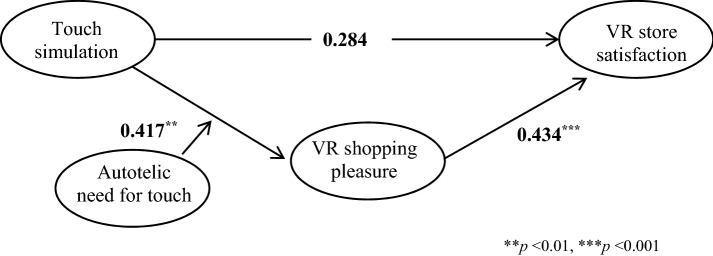
Moderating model of autotelic need for touch

Further, the moderating effect of autotelic NFT was examined (H7b). The moderating role of autotelic NFT between touch simulation and VR shopping pleasure was analyzed via the PROCESS Macro Model 7 used with 5,000 bootstrap samples. VR shopping time was controlled as a covariate. As shown in Fig. 4, the interplay effect of touch simulation and autotelic NFT on VR shopping pleasure was statistically significant (*b* = 0.417, 95% CI [0.149, 0.686]). According to these results, in the case of high autotelic NFT (Mean + 1*SD*), touch simulation significantly influenced VR shopping pleasure (Effect = 0.462, 95% CI [0.145, 0.780]) (see Table 3); however, when autotelic NFT was medium or low level (Mean–1SD), the effect of touch simulation on VR shopping pleasure was not significant. The indirect effect of touch simulation on VR store satisfaction through VR shopping pleasure was also significant (the index of moderated mediation = 0.181, 95% CI [0.027, 0.357]) only for individuals with a high level of autotelic NFT (Effect = 0.201, bootSE = 0.095, 95% CI [0.038, 0.406]) (see Table 4). This pattern of results was consistent with H7b.

**Table 3:** Conditional effects of touch simulation on pleasure at values of autotelic need for touch

Autotelic need for touch	Effect	BootSE	95% Confidence Interval	
			LLCI^a^	ULCI^b^
Mean – 1*SD* (2.600)	− 0.288	0.172	− 0.634	0.057
Mean (3.600)	0.128	0.113	− 0.097	0.355
Mean + 1*SD* (4.400)	0.466	0.158	0.145	0.780

^a^LLCI: lower limit confidence interval

^b^ULCI: upper limit confidence interval

**Table 4:** Conditional indirect effects of touch simulation on VR store attitude at values of autotelic need for touch

Autotelic need for touch	Effect	BootSE	95% Confidence Interval	
			LLCI^a^	ULCI^b^
Mean – 1*SD* (2.600)	− 0.125	0.077	− 0.282	0.016
Mean (3.600)	0.056	0.043	− 0.022	0.151
Mean + 1*SD* (4.400)	0.201	0.095	0.038	0.406

^a^LLCI: lower limit confidence interval

^b^ULCI: upper limit confidence interval

## Discussion

We explored the psychological mechanism driving consumers’ satisfaction in shopping in a VR store. To address the gap in the research on the vicarious touch sensory in the VR environment, we examined the role of touch simulation on VR store satisfaction. We found that being able to imagine touching a product can positively influence consumers through VR shopping self-efficacy and VR shopping pleasure. In particular, the role of VR shopping self-efficacy found in the current study was in line with the concept of Technology Acceptance Model (Ajzen & Fishbein, 1980), where individuals’ acceptance of a certain technology was based on their ability and probability of performing a particular task or behavior (i.e., perceived ease-of-use). The role of VR shopping self-efficacy was also observed in a relationship with VR shopping pleasure. Specifically, touch simulation could influence the pleasure of shopping at the VR store only through VR shopping self-efficacy; the direct path from touch simulation to VR shopping pleasure was insignificant, but the indirect path through VR self-efficacy connected touch simulation and pleasure, leading to VR store satisfaction. This indirect effect can be explained by the challenge of the VR technology adoption that the vicarious touch that consumers experience in the current study was insufficient to generate pleasure, and only those who feel comfortable and confident in using the technology was able to feel pleasure using the technology (Compeau & Higgins, 1995; Kulviwat et al., 2014). However, note that the significance of the indirect path was marginal, that is, finding nonsignificant indirect relationships in other settings is possible; even with high self-efficacy in using the VT technology, the vicarious touch store may not be enough to create a feeling of pleasure in the VR store.

Regarding the moderating effect of NFT, the independent effect of instrumental and autotelic NFT was tested, which drew a distinction between the roles of autotelic and instrumental NFT (Atakan, 2014; Peck & Childers, 2003; Peck & Wiggins, 2006; Peukert et al., 2019; Ranaweera et al., 2021). We found that the effect of touch simulation on VR shopping pleasure was moderated by autotelic NFT. This could be because individuals with high autotelic NFT, who use tactile information as a source of pleasure, can feel pleasure easily when vicarious touch experience is activated in a VR environment. Simulated touch feeling in the VR store might elicit perceived pleasure only for individuals with high autotelic NFT. However, the effect of touch simulation on VR shopping self-efficacy was not moderated by instrumental NFT. Even though individuals with high instrumental NFT have utilitarian motivations for touch (Peck & Childers, 2003), they did not influence the utilitarian path between touch simulation and VR shopping self-efficacy. This could be because the stimulated touch feeling may not provide sufficient immersive experience to drive VR shopping self-efficacy in individuals with high instrumental NFT to meet their utilitarian and purposeful needs for touching a product (Peck & Childers, 2003).

## Conclusions

This study makes several academic contributions. First, the role of touch simulation in a VR environment was extended. Although the concept of mental simulation is inferred as a factor inducing consumer responses in a VR shopping environment, the focus has been on its formation from a vivid image or based on information (MacInnis & Price, 1987; Percy & Rossiter, 1983; Song & Kim, 2012); to fill the void, this study explored the touch simulation activated by individuals’ action in the context of a VR experience. These findings contribute to expanding the literature on the VR effects on consumer responses in the retail industry by showing the simulated touching effect on consumer satisfaction with the store. Second, this study demonstrated the mediating role of VR shopping self-efficacy and VR shopping pleasure on VR store satisfaction. While relatively little research has focused on the antecedents and mediators of self-efficacy in technology adoption, this study presented how self-efficacy can be enhanced in an unfamiliar and technically challenging environment, such as a VR store. Touch simulation was found to be a significant factor in increasing consumers’ VR shopping self-efficacy. In addition, after clarifying the relationship between touch simulation and self-efficacy empirically in a virtual retail context, this study identified the marginal but significant mediating role of pleasure that connects consumer’s self-efficacy to satisfaction. The increased VR shopping self-efficacy positively influences VR store satisfaction through touch simulation and VR shopping pleasure successively. Third, this study verified the moderating effect of autotelic NFT between touch simulation and VR shopping pleasure. Several studies found that positive emotions such as fun, pleasure, and enjoyment are important factors that lead to positive consumer responses (Steuer, 1992; Weibel et al., 2008). However, only a few studies have explored when and how pleasure was perceived in a VR environment. This study demonstrated that touch simulation can be a factor that drives pleasure for people with high autotelic NFT. This study, therefore, adds to the literature on VR experience by identifying a new antecedent of VR shopping pleasure. Fourth, this study contributes to the diversification of data collection methods in VR studies. VR stimuli with head-mounted devices were used in this study to provide an immersive VR store experience in a laboratory environment. Numerous VR-related studies adopt non-immersive VR, which uses a flat screen (Lee & Chung, 2008; Li et al., 2002; Shin & Shin, 2011; Suh & Lee, 2005). Unlike research with non-immersive VR, the findings of the current research were obtained from participants’ actual experience in an immersive VR store by wearing head-mounted devices. Finally, this study suggests managerial implications for the consumer experiences of VR stores. Considering the importance of touch simulation through self-efficacy, retailers should focus on delivering strong touch sensory experiences, even with vicarious touch experiences, that can enhance self-efficacy in using VR technology. When the technology is limited in providing strong touch simulation, retailers may focus on educating consumers to boost their self-efficacy with the use of VR technology and on reducing the perceived difficulty of the technology because their increased VR self-efficacy can positively influence VR store satisfaction. Although the study makes several contributions, it also has limitations. First, given the nature of the laboratory study, which requires one participant to be examined at a time, the number of data used herein can be a limitation. Future studies must use a sample size large enough to provide better statistical power. Second, the results from this study may be difficult to generalize to consumers of different ages because only individuals in their 20s and 30s participated in this study. Thus, future research using samples from different ages or characteristics would be worthwhile for generalization. In addition, future research could verify moderating variables other than autotelic and instrumental NFT to understand the psychological mechanism driving consumers in a VR environment. In particular, individuals’ experience or knowledge about VR stores or advanced technology could be applied as moderators. As individual NFT is an innate characteristic, retailers or marketers cannot control it. However, prior VR store experience or related knowledge can be expanded through marketing programs or other sources of information. Thus, additional studies exploring other moderators that can be controlled by retailers or marketers will be useful for practical implications.

## Data Availability

The datasets used and/or analyzed during the current study are available from the corresponding author on reasonable request.
